# Temporally Prescription of Care: a Challenge to Advance in Nursing Sciences

**DOI:** 10.17533/udea.iee.v42n2e01

**Published:** 2024-07-03

**Authors:** Loreto Maciá Soler

**Affiliations:** 1 Nurse, PhD. Professor, Universidad de Alicante (Spain). Email: loreto.macia@gcloud.ua.es Universidad de Alicante Universidad de Alicante Spain loreto.macia@gcloud.ua.es

Within the Nursing field, it is common that when talking about prescription an association is established among drug administration, prescribed dosage, frequency and route of administration and it is also normal for this to be the case; a drug without dosage, frequency and route of administration is not of much use, given that little could be decided about its effects, safety in administration, and response of the body to substances prescribed to solve health problems. Following this common thread, when we nurses hear about prescription, we quickly associate this term with drugs, opening a collective debate between what can be prescribed and what cannot. However, we often forget our real competence in prescribing, which is undoubtedly care.

Prescribing care is complex, especially if it is done well and a correct prescription if it does not include temporality or, what amounts to the same; how often should care be performed for it to be effective? It cannot be considered a prescription of care because a cause-effect relationship cannot be established between the prescribed care and the result obtained for the person. The temporary prescription of care has three elements: (i) Statement of the problem to be treated, the diagnosis of which is obtained after assessing the person object of care. This element derives from nursing's own competencies and its responsibility for healthy or sick individuals;^1^ (ii) Timing of application of the prescribed care that allows the expected effect to be measured/evaluated. This element is related with the quality of care and with patient safety that will permit quantifying the cost and effectiveness of the care;^2^ and (iii) Care order issued by a nursing professional to resolve identified problems. This element is linked to leadership and responsibility in care, as well as decision making with legal support.^1^

With these three elements, the quality of care can be evaluated, or in other words, its effect on people. If any of the three is missing, we cannot evaluate the quality of care and, hence, a gap will remain in the care required by patients, which is the care they must receive upon the onset of a health problem regarding the system of care administered during a nursing work shift. The difference between the care required and the care administered defines the number of nurses needed in a care unit. Without using the three prescription elements, we know little about patient safety, exercise of own competencies, and nursing decision making. With the three elements cited and using the “temporally prescription of care” we solve important aspects of care, NCP. If, additionally, we include this concept in the nursing care process (NCP) as work method, we convert the NCP into a useful quality care tool to calculate nursing ratios and decision making about patient care, which are clinical safety elements.

One might think that attributing all these benefits to temporally prescription is an exaggeration; however, it is not. I will try to explain it briefly. As an example, we will place ourselves in a hospitalization ward of an acute care hospital; that is with surgical and non-surgical processes, and patients with a mean stay between 7 and 9 days. The nurse responsible for the patient and, therefore, for the effects of hospitalization, performs the daily assessment of the patient from admission to discharge throughout the care process and conducts said evaluation with a method that will permit evaluating the quality of care to solve health problems identified during hospitalization.

Each day, the nurse will prescribe the care required by the patient assessed according to the problems identified, for which it is advisable to use language understood by the entire health staff because health care is not provided alone, but rather within a health team. It is advisable to use internationally recognized language ​​to define problems and issue care orders, given that understanding orders will facilitate their execution and enhance the activities carried out within the evolution of the patients. (Element 1)

The next step is temporality; here, we introduce a care quality term that is time in the form of how often must we perform an activity prescribed for each patient. (Element 2) 

Actually, if we have no evidence of the effect of mobility, feeding, hygiene, or rest as preventive elements of adverse effects that occur during hospitalization, it is quite difficult to compare the expected result with the prescribed indication (recall the medication example) and this care, related with autonomy and comfort, intervenes directly upon adverse effects of hospitalization, such as infections, falls, or onset of ulcers, which are the responsibility of medical and nursing professionals according with the World Health Organization.^3^

Finally, we will issue the care order that must be administered for each patient (Element 3) so that its effectiveness can be evaluated and that, when those responsible for management require criteria for assigning staff, costs, quality of care, they can find this agile, truthful, and accessible information. To measure the effectiveness of care, we must ask and answer the following questions:

Why is care conducted? Because a nurse prescribes it after assessing a patient's situation during hospitalization regarding care needs.

How is it conducted? In accordance with the use of manual guide protocols that are updated, accessible and which bear valid evidence.

How often must this care be carried out? (temporality) and what result is expected.^4^

How is the patient’s evolution monitored? Regarding monitoring and, merely as an example, if 24 hours after prescribing care, we fail to evaluate its effectiveness, we will not know anything about the result of the prescription or its quality.^4^

Prescribing an activity does not necessarily mean directly executing it. If an activity related to hygiene or feeding, devices or clothing is prescribed, it is not necessary for highly trained nurses to assume such directly, as long as the staff has qualified support personnel. It is about assessing the staff’s competence and issuing a care order that, thereafter, we will verify as performed in addition to providing documentary evidence of the execution of the task. A nursing responsibility involves verifying the effectiveness of the activities conducted and which were ordered, to advance the prescription, modifying if necessary, the temporality of the prescribed care and eliminating or adding other care activities.

Issuing orders is a function of any profession responsible for patients and, in this case, medical professionals are responsible for the admission and discharge of patients in hospitalization units and nurses are responsible during hospitalization to make sure no adverse effects occur and that people admitted to a hospital are safe, besides to complying with the treatment prescribed by medical professionals.

When we administer a medication or perform an invasive procedure derived from disease diagnosis and treatment, maximum safety must be guaranteed and when care is prescribed autonomously, it must also be done with the maximum knowledge and the greatest evidence available. All this without fear of giving orders and with the professional attitude of knowing how to receive them.

The prescription of basic, advanced, and technical care on a temporal basis in medical-surgical hospitalization units currently represents a gap in care plans; nursing students are acquiring the competence of responsibility for basic care that is typical of nursing from the hands of a professional group that does not have decision-making power over care; thereby, generating a somewhat confusing situation in the knowledge and application of basic care.^4^

In the European Union, the legal support for competencies related to care is included both in the state orders regulating Nursing curricula and in the community directive on regulated professions, which includes, along with Nursing, the professions of Midwifery, Dentistry, Medicine, Pharmacy and Veterinary Medicine. In said directive ^1^, The competencies conferred by the title of Nurse responsible for general care are: (i) Competence to independently diagnose necessary nursing care using theoretical and clinical knowledge, and to schedule, organize, and manage nursing care when treating patients based on the knowledge and skills acquired during training; (ii) Competence to effectively collaborate with other parties from the health sector, including participation in the practical training of health staff about the base of knowledge and skills acquired; (iii) Competence to hold individuals, families, and groups responsible for healthy lifestyle habits and health care based on the knowledge and skills acquired; (iv) Competence to, independently, take immediate measures to maintain life and apply measures in crisis and catastrophic situations; (v) Competence to, independently, grant advice and instructions and provide support to those who need care and to their loved ones; (vi) Competence to, independently, guarantee the quality of nursing care and evaluate such; (vii) Competence to establish full professional communication and cooperate with members from other health-sector professions; and, (viii) Competence to analyze the quality of care and improve their own professional practice as nurses responsible for general care.

In the community directive, clear competencies may be observed, which support the clinical decision-making of nurses and not solely in the field of hospitalization but also in primary health care.^5^ It seems obvious that making decisions about care and prescribe temporally the activities necessary to guarantee quality of care is a component that strengthens its own portfolio of services, from which related lines of research emerge that allow identifying the ideal temporality required for the application of care..

Research questions, like how often should a hospitalized person be moved, involve delving further into nursing knowledge that lacks evidence, ensuring the quality of care related in this case to mobilization, improving people's comfort, and advancing Nursing Sciences within our social space, which is maintaining which is maintaining the autonomy of people when they get sick, preventing disease, and accompanying them when life ends.
